# INVESTIGATING HEALTHCARE ACCESS DISPARITIES IN MINNESOTA THROUGH GIS ANALYSIS

**DOI:** 10.1093/geroni/igae098.4321

**Published:** 2024-12-31

**Authors:** Woo Jang, Drew Krull, Seonhwa Lee, Merril Silverstein

**Affiliations:** Minnesota State University, Mankato, Mankato, Minnesota, United States; ERM, Minneapolis, Minnesota, United States; Texas Women’s University, Denton, Texas, United States; Syracuse University, Syracuse, New York, United States

## Abstract

According to the 2020 report on population projections from the Minnesota State Demographic Center, older residents aged 85 and above are expected to more than double within the next 35 years. The report also highlights that in 2033, 32% of rural counties will experience a higher proportion of residents aged 65 years and older than 19% for urban counties. With the expected increase in the older population, the demand for healthcare services in rural areas will substantially rise. However, healthcare facilities are more dispersed in rural areas. Despite several accounts pointing to healthcare-deprived areas or the differential geographic presence of healthcare resources in recent years, GIS-based analysis in this area remains limited. To address this gap, our study utilizes data from the 2020 Census to assess various socio-demographic and economic factors shaping healthcare access inequalities for individuals aged 65 years and older in Minnesota. Employing GIS techniques, we identify unique spatial patterns and associated social factors contributing to healthcare access disparities. Our results visualize detailed maps highlighting areas facing challenges in accessing healthcare services and low-access populations across Minnesota. This study provides insights into understanding the spatial and social determinants of healthcare disparities, which assist policymakers and healthcare professionals to allocate resources more efficiently and address the unique needs of the target group.

